# Supplementary material for: A carboxy-terminal ubiquitylation site regulates androgen receptor activity
Source: Commun Biol. 2024 Jan 5;7:25. doi: 10.1038/s42003-023-05709-x (PMC10770046; doi:10.1038/s42003-023-05709-x)
Supplement: Supplementary file 2 — Supplementary Information [file 42003_2023_5709_MOESM2_ESM.pdf]

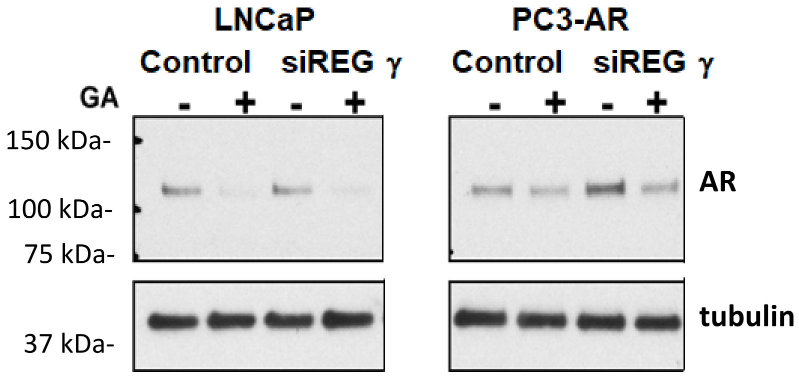

Supplementary Figure 1. AR degradation is not REGγ dependent. Cells were transfected with control or REGγ siRNA for 2 days in complete medium. They were then cultured for 24 hrs in medium with vehicle or geldanamycin (GA), followed by immunoblotting.

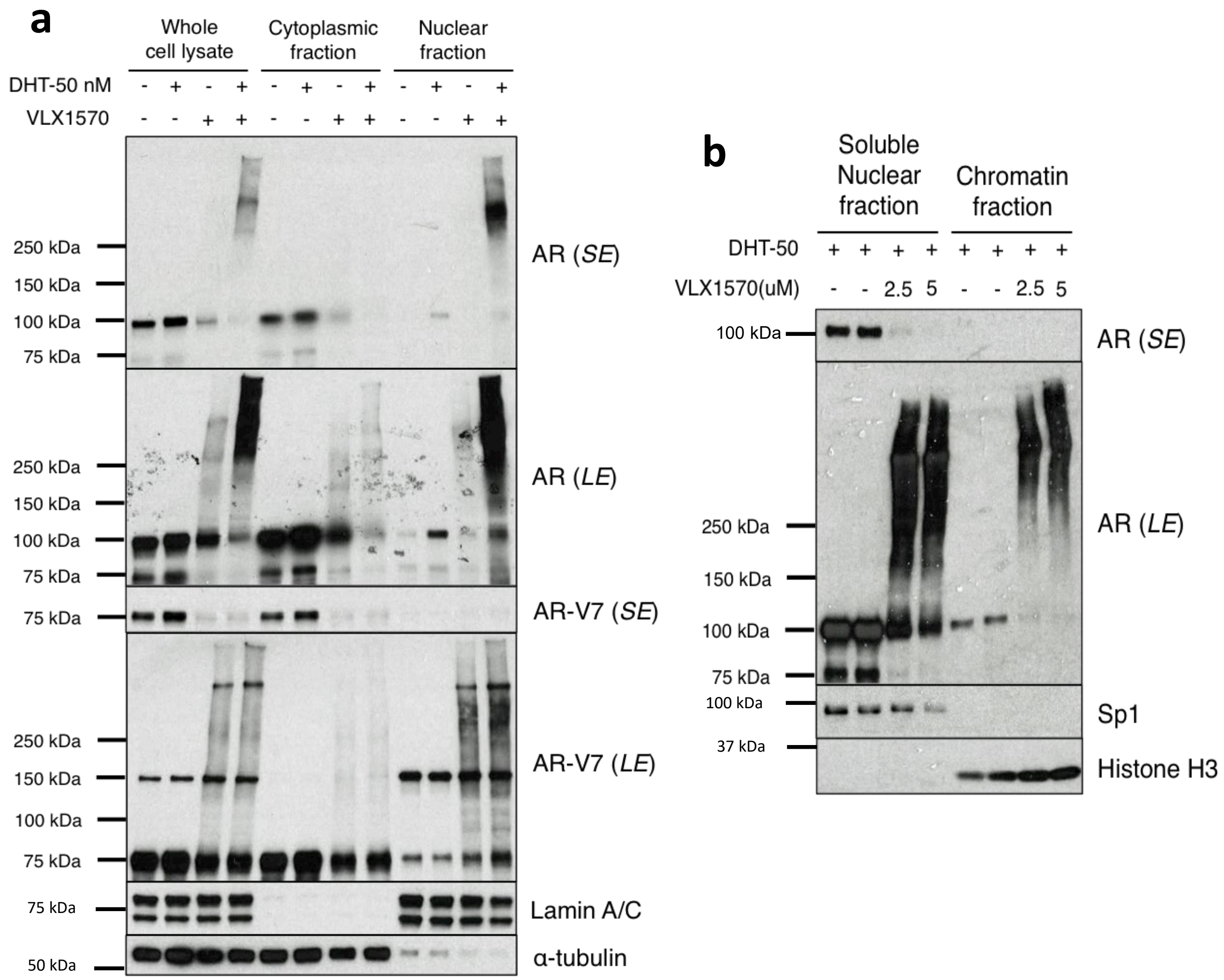

Supplementary Figure 2. VLX1570 induced high molecular weight AR species are primarily nuclear. (a) VCaP cells in CSS medium were treated for 6 hrs with DHT (50 nM) and/or VLX1530 (5 μM). Whole cell lystaes, cytoplasmic, and nuclear fractions were then isolated, and equal amounts of total protein were immunoblotted. **(b)** VCaP cells treated as in (A) were separated into soluble and chromatin bound nuclear fractions and immunoblotted. Each blot shows long exposures (LE) and short exposures (SE) for better assessment of AR and AR-V7 levels.

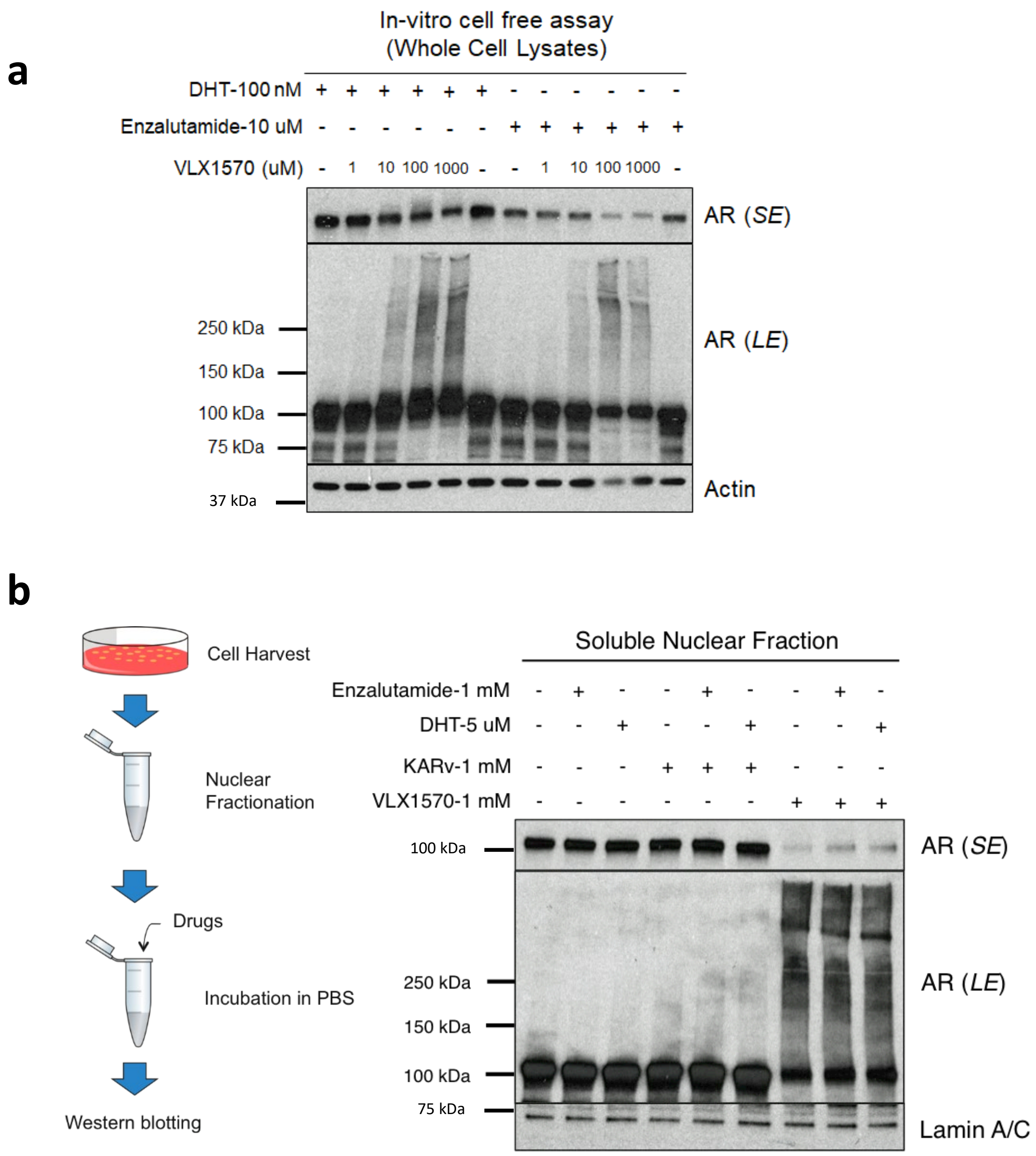

Supplementary Figure 3. VLX1570 generates high molecular weight AR species in cell free extracts. (a) Whole cell lystaes were generated from VCaP cells cultured for 24 hrs in medium with DHT or enzalutamide. Lysates were then incubated with VLX1570 for 30 minutes and immunoblotted for AR. **(b)** Soluble nuclear fraction was isolated from VCaP cells grown in complete FBS medium. Aliquots were then incubated with indicated drugs for 30 minutes and immunoblotted. (KARv-1 is a CDK9 inhibitor). Long exposures (LE) and short exposures (SE) are shown.

MS/MS Fragmentation of ALLLFSIIPVDGLKNQK
Found in ANDR_HUMAN in Human, Androgen receptor OS=Homo sapiens OX=9606 GN=AR PE=1 SV=3

Match to Query 48347: 1983.188206 from(992.601379,2+) intensity(324150.4922) rtinseconds(4154.6686) index(24485)
Title: 122812RZYDHTMGUP.9523.9523.2 File:"122812RZYDHTMGUP.raw", NativeID:"controllerType=0 controllerNumber=1 scan=9523"
Data file C:\ProgramData\Matrix Science\Mascot Daemon\MGF\1601 S Balk AR PTM Human combine Rerun\mascot_daemon_merge.mgf

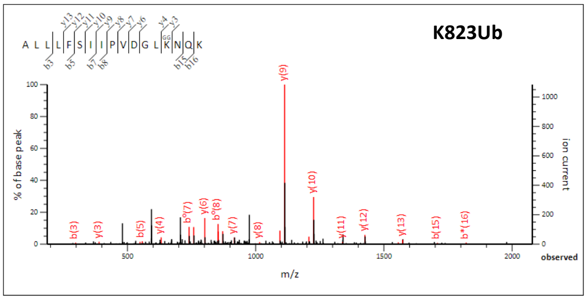

Score	Mr(calc)	Delta	Sequence	Site Analysis
37.3	1982.1564	1.0318	ALLLFSIIPVDGLKNQK	GG K14 63.31%
35.0	1982.1564	1.0318	ALLLFSIIPVDGLKNQK	GG K17 36.69%

MS/MS Fragmentation of NQKFFDELR
Found in ANDR_HUMAN in Human, Androgen receptor OS=Homo sapiens OX=9606 GN=AR PE=1 SV=3

Match to Query 37303: 1309.641576 from(655.828064,2+) intensity(7408540.8477) rtinseconds(2479.571) index(39500)
Title: 081712RZYupper.6072.6072.2 File:"081712RZYupper.raw", NativeID:"controllerType=0 controllerNumber=1 scan=6072"
Data file C:\ProgramData\Matrix Science\Mascot Daemon\MGF\1601 S Balk AR PTM Human combine Rerun\mascot_daemon_merge.mgf

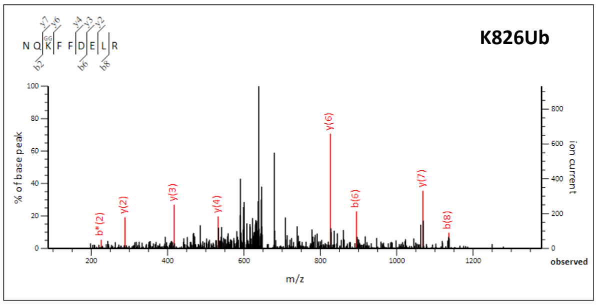

MS/MS Fragmentation of FYQLTKLLDSVQPIAR
Found in ANDR_HUMAN in Human, Androgen receptor OS=Homo sapiens OX=9606 GN=AR PE=1 SV=3

Match to Query 48621: 2006.118138 from(1004.066345,2+) intensity(418751.0303) rtinseconds(3836.0981) index(23912)
Title: 122812RZYDHTMGUP.8783.8783.2 File:"122812RZYDHTMGUP.raw", NativeID:"controllerType=0 controllerNumber=1 scan=8783"
Data file C:\ProgramData\Matrix Science\Mascot Daemon\MGF\1601 S Balk AR PTM Human combine Rerun\mascot_daemon_merge.mgf

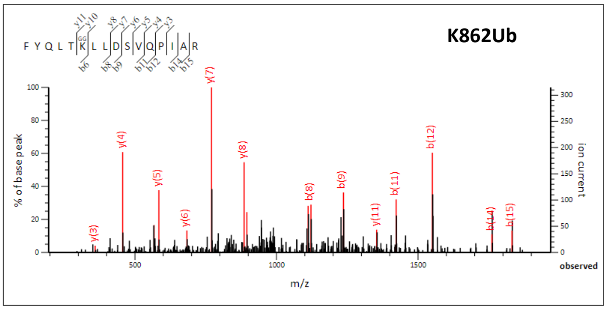

MS/MS Fragmentation of ILSGKVKPIYFHTQ
Found in ANDR_HUMAN in Human, Androgen receptor OS=Homo sapiens OX=9606 GN=AR PE=1 SV=3

Match to Query 44953: 1744.990942 from(873.502747,2+) intensity(466571.7891) rtinseconds(2366.9788) index(4146)
Title: 122812RZYMGUP.5351.5351.2 File:"122812RZYMGUP.raw", NativeID:"controllerType=0 controllerNumber=1 scan=5351"
Data file C:\ProgramData\Matrix Science\Mascot Daemon\MGF\1601 S Balk AR PTM Human combine Rerun\mascot_daemon_merge.mgf

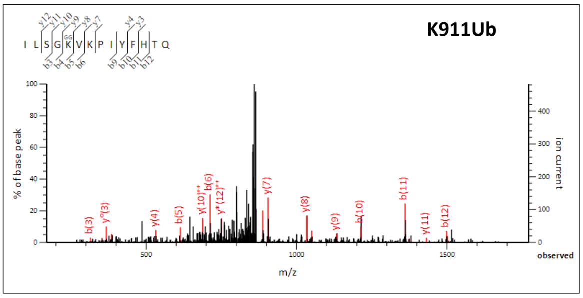

Score	Mr(calc)	Delta	Sequence	Site Analysis
33.8	1743.9672	1.0238	ILSGKVKPIYFHTQ	GG K5 97.98%
17.0	1743.9672	1.0238	ILSGKVKPIYFHTQ	GG K7 2.02%

Supplementary Figure 4. Identification of AR ubiquitylation sites. MS/MS HCD spectra for peptides containing the GlyGly (GG) ubiquitin remnant are shown. Two of the peptides had more than one lysine. The ubiquitylation site probability for the K823 site is ~63% probability, with ~37% probability of the ubiquitylation being on K826. The ubiquitylation site probability for the K911 site was ~98% probability, with K913 being much lower probability (~2%).

a

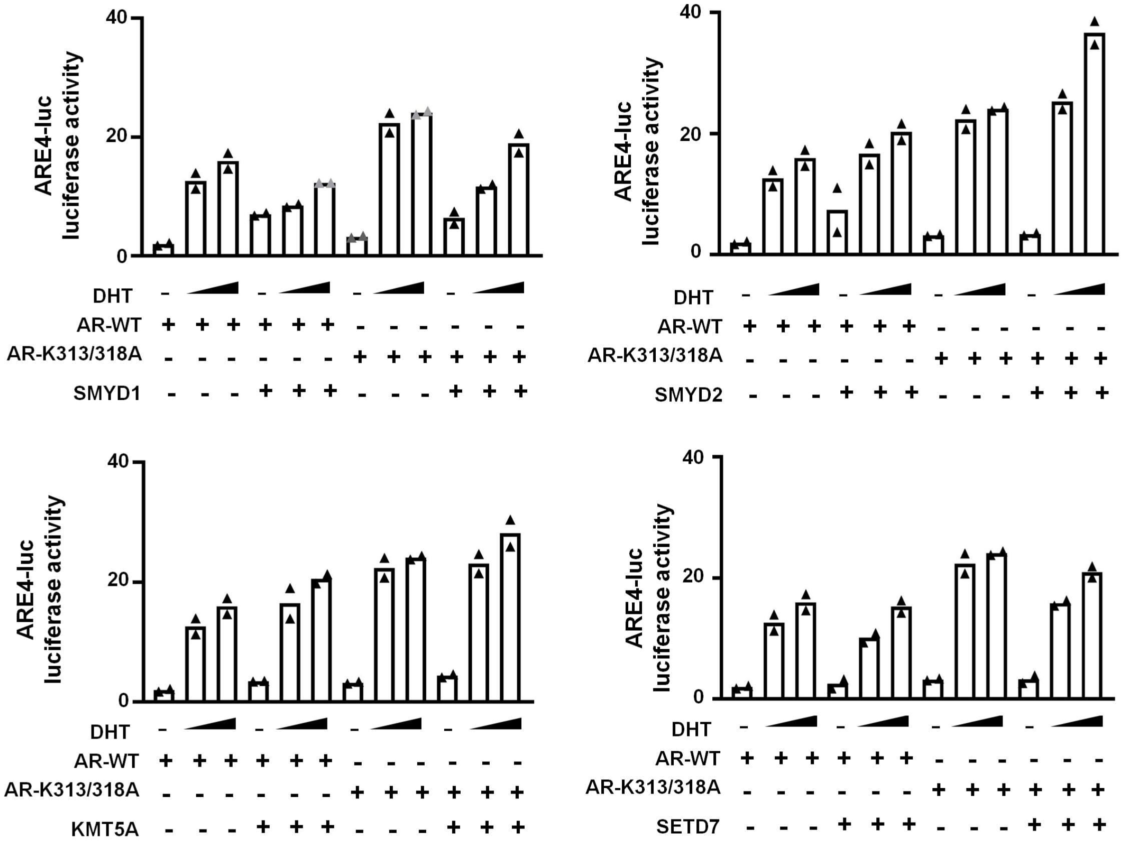

b

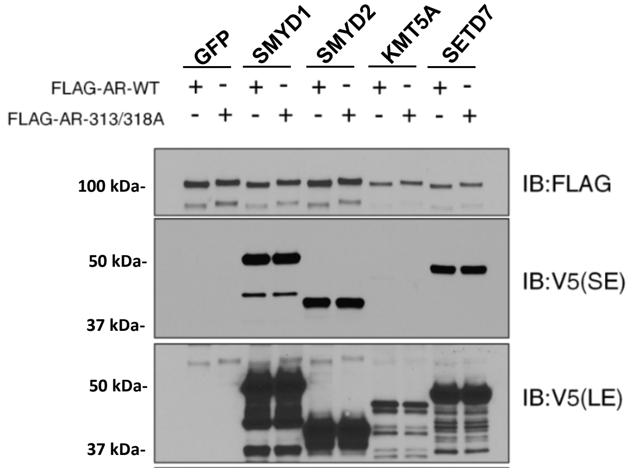

Supplementary Figure 5. AR K313/318 mutations do not alter responses to histone methyltransferases. (a) Wild-type or K313/318A mutant ARs (Flag tagged) were transiently expressed for 2 days in COS7 cells with an ARE-luciferase reporter and the indicated methyltransferases (V5 tagged). Cells were then cultured for 24 hrs in CSS medium with 0, 1, or 10 nM DHT, and luciferase activity was measured. Data are mean of biological replicate samples. **(b)** Lysates from cells in (A) that did not get DHT were immunoblotted for Flag (AR) and for V5 (methyltransferase).

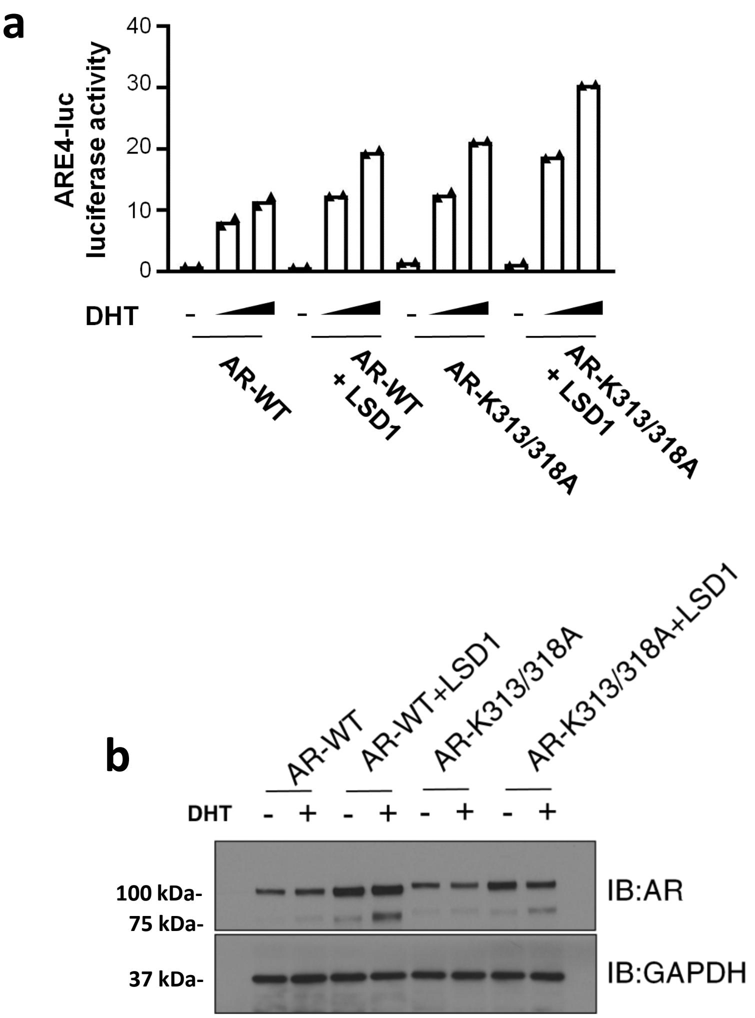

Supplementary Figure 6. AR K313/318 mutations do not alter responses to LSD1. (a) Wild-type or K313/318A mutant ARs (Flag tagged) were transiently expressed for 2 days in COS7 cells with an ARE-luciferase reporter and LSD1 (V5 tagged). Cells were then cultured for 24 hrs in CSS medium with 0, 1, or 10 nM DHT, and luciferase activity was measured. Data are mean of biological replicate samples. (**b**) Lysates from cells in (a) were immunoblotted for Flag AR.

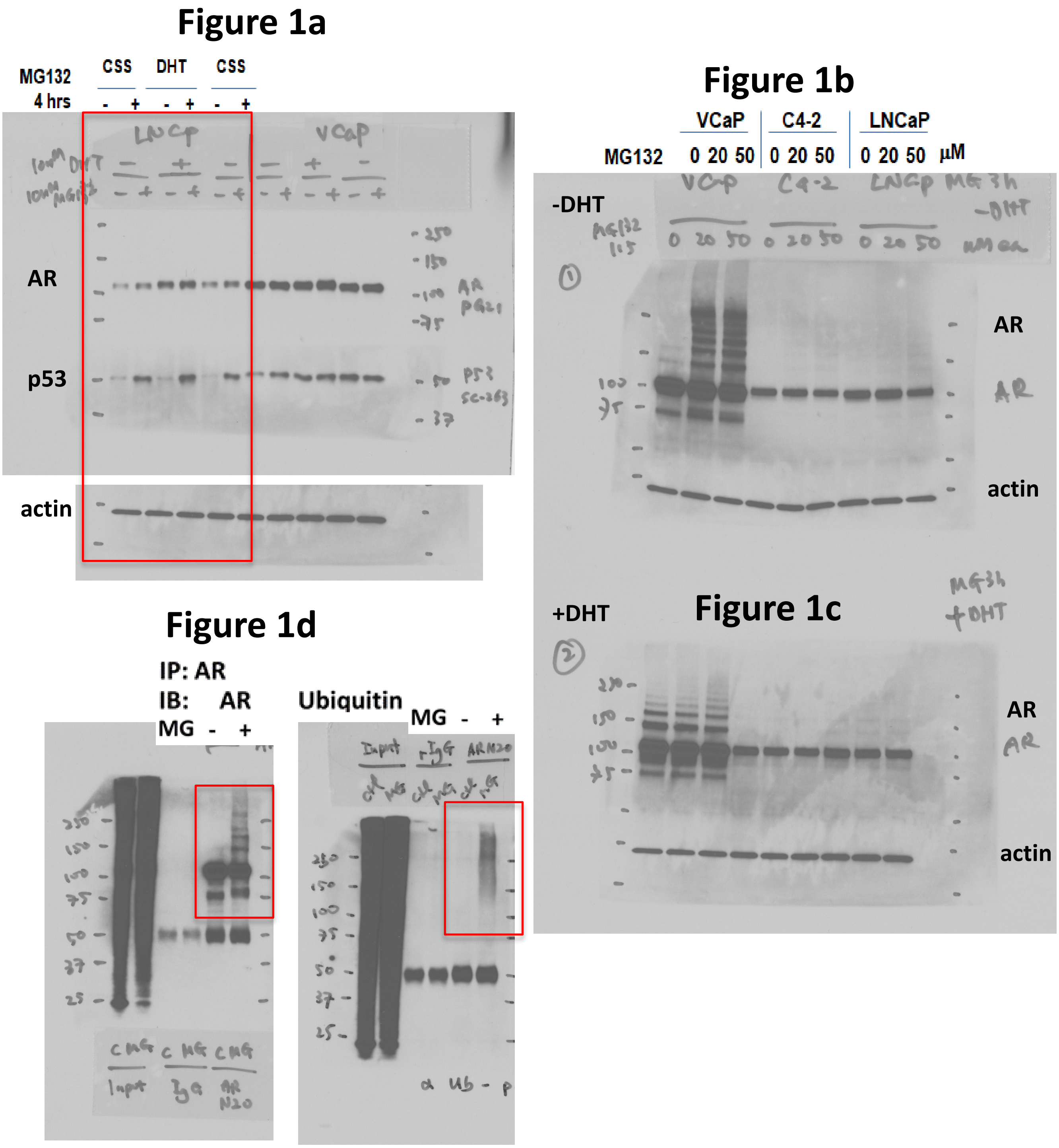

Supplementary Figure 7. Gels corresponding to Figure 1. Portions of the gel shown in the figure are highlighted. Molecular weights were determined by overlaying autorads onto membranes with colored markers and marking band positions.

Figure 2a

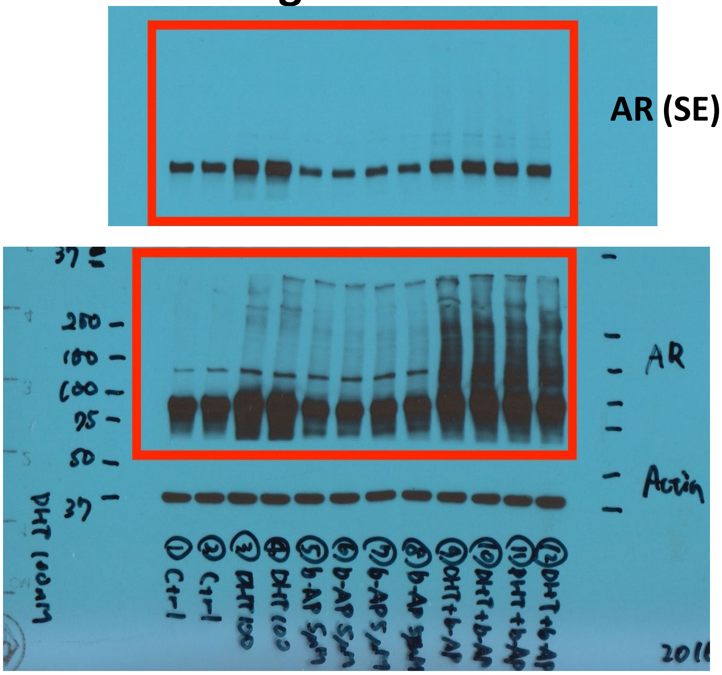

Figure 2b

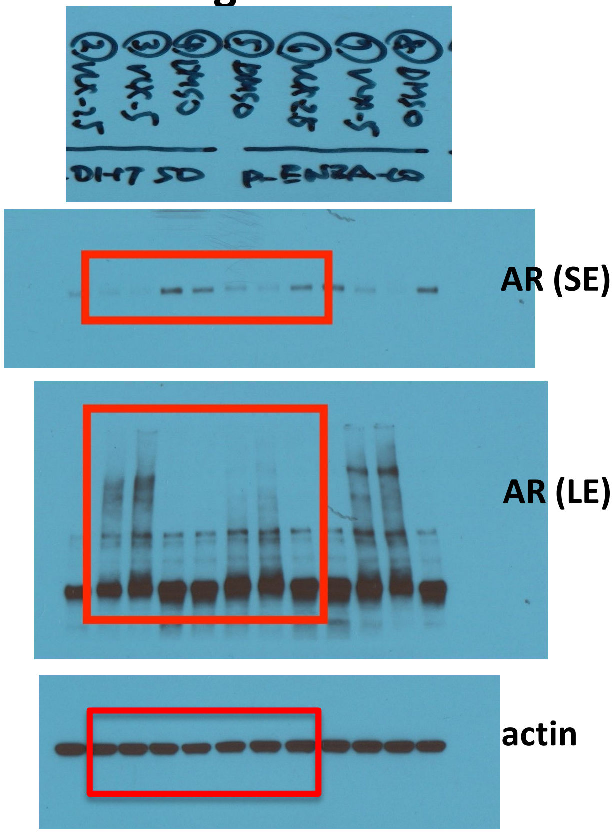

Figure 2c

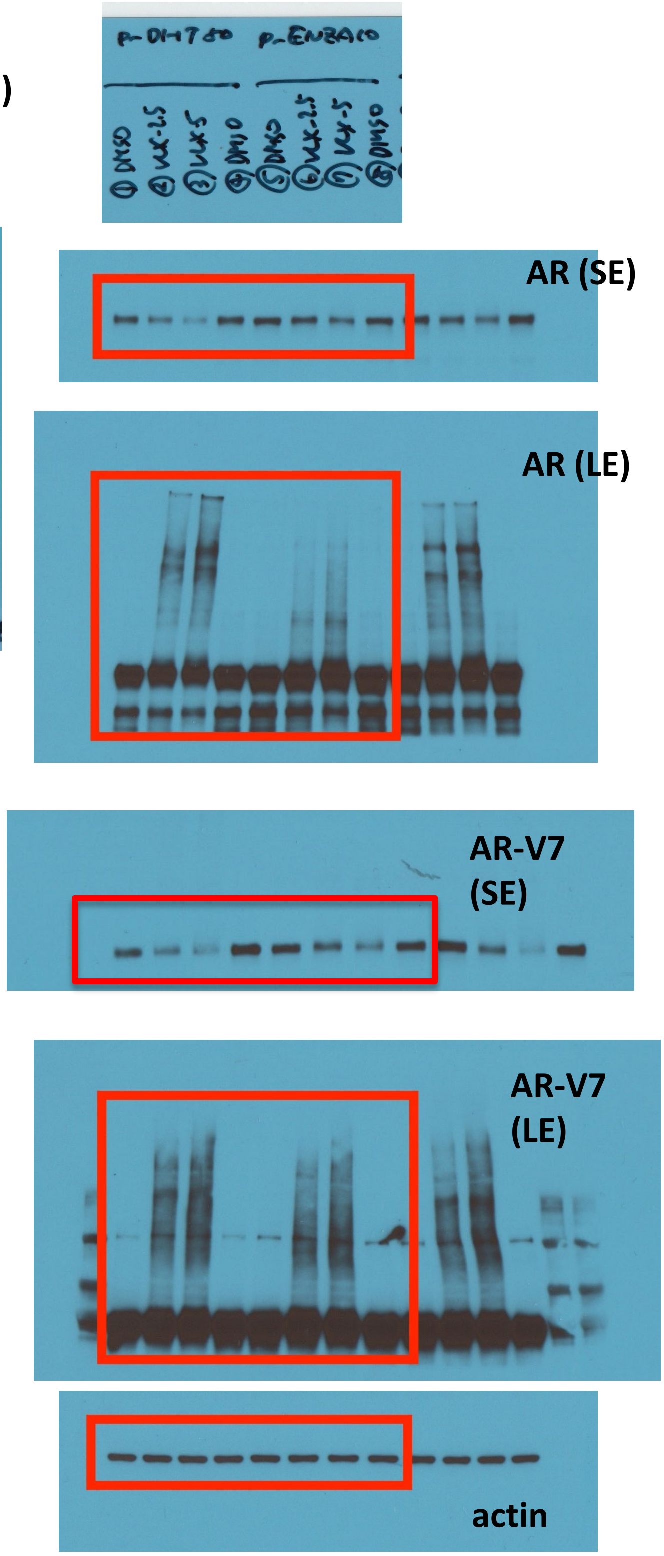

Supplementary Figure 8. Gels corresponding to Figure 2. Portions of the gel shown in the figure are highlighted. Molecular weights were determined by overlaying autorads onto membranes with colored markers and marking band positions.

Figure 3a

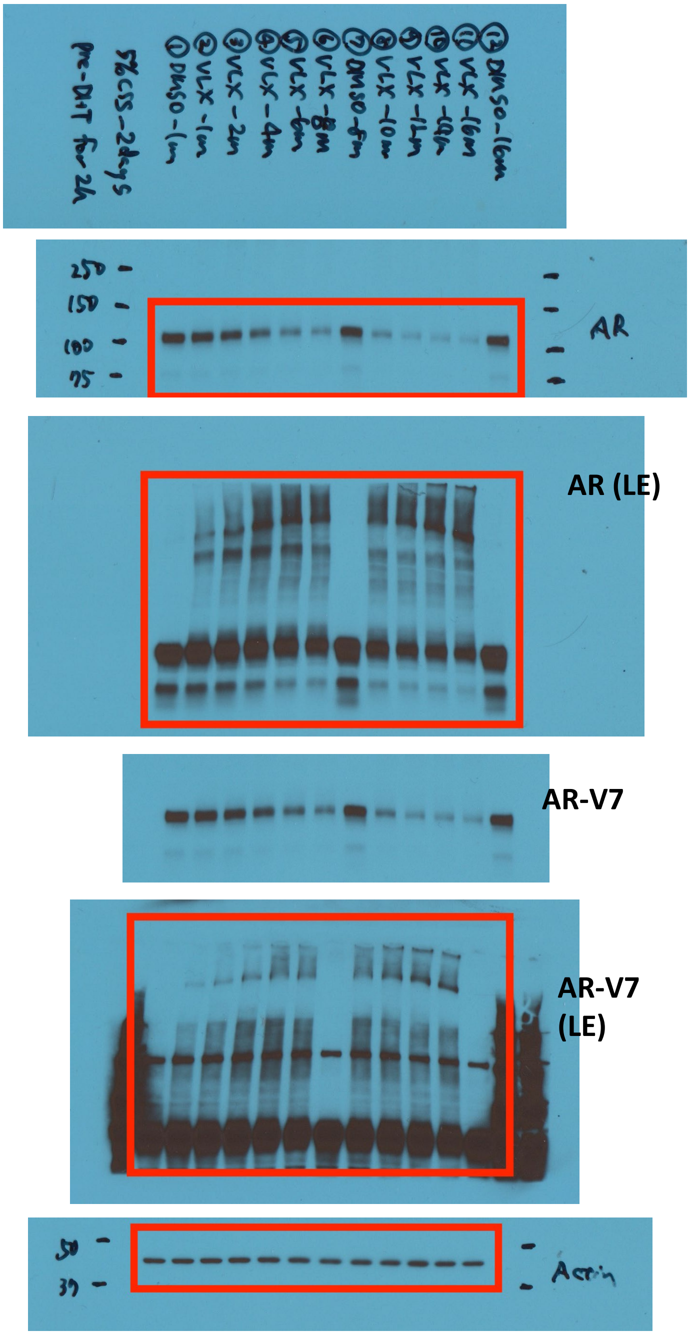

Figure 3b

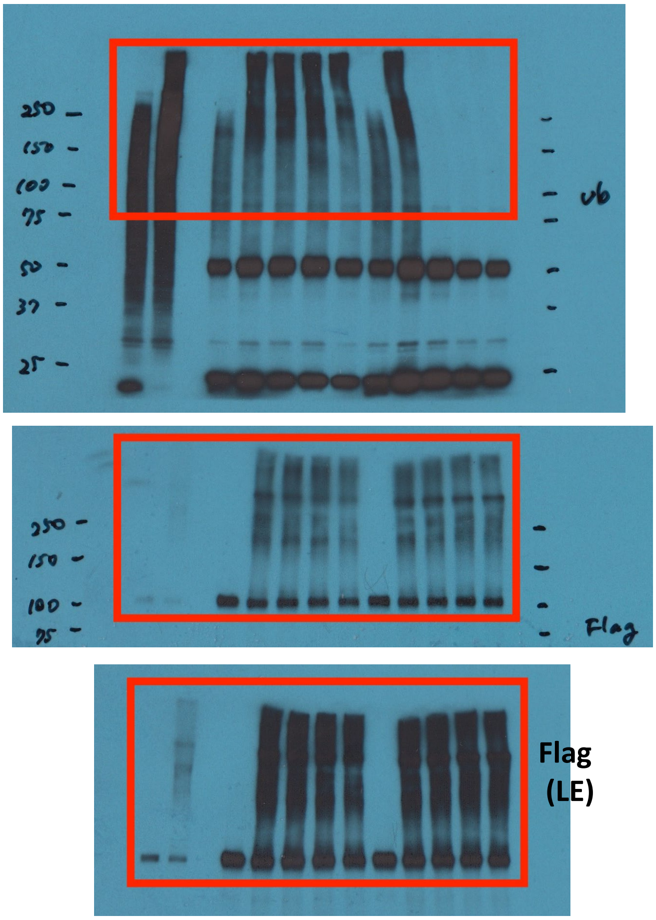

Supplementary Figure 9. Gels corresponding to Figure 3. Portions of the gel shown in the figure are highlighted. Molecular weights were determined by overlaying autorads onto membranes with colored markers and marking band positions.

Figure 4a

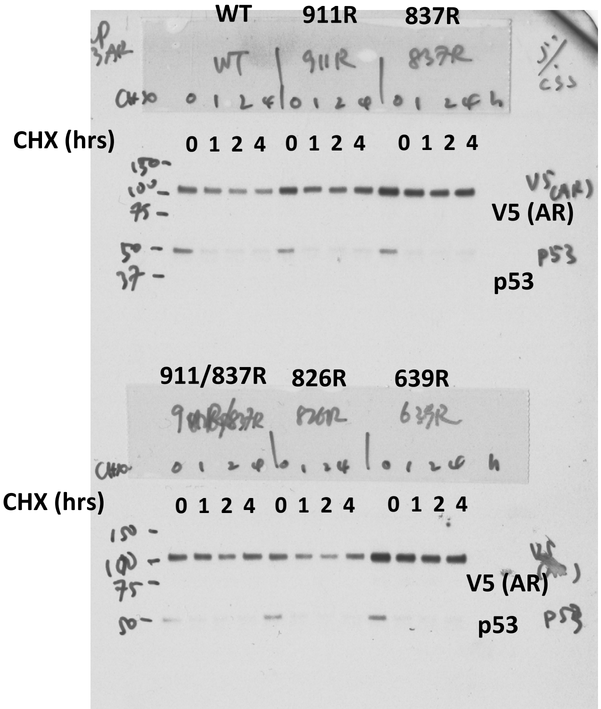

Figure 4b

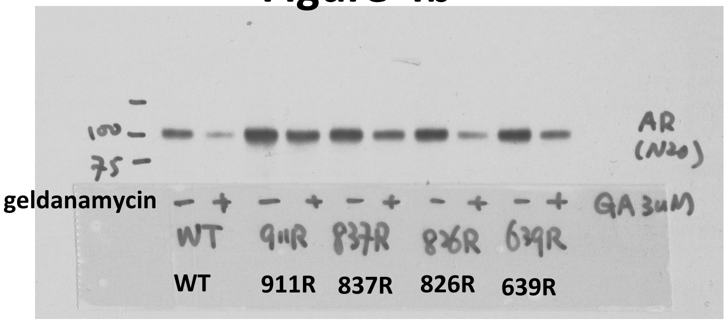

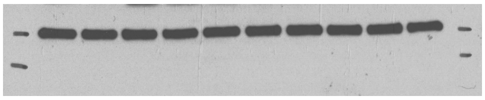

Figure 4c

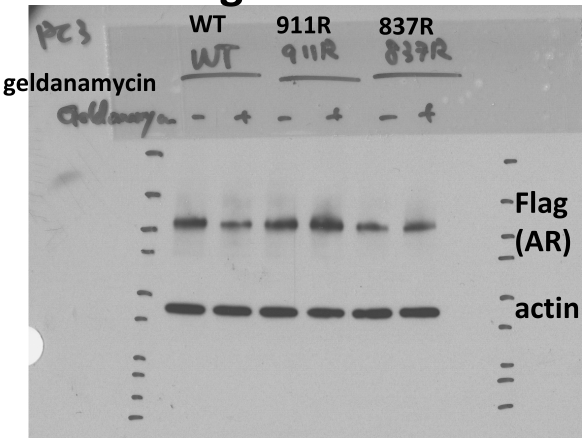

Figure 4d

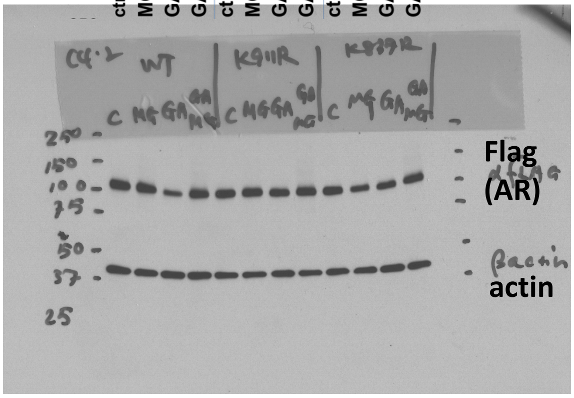

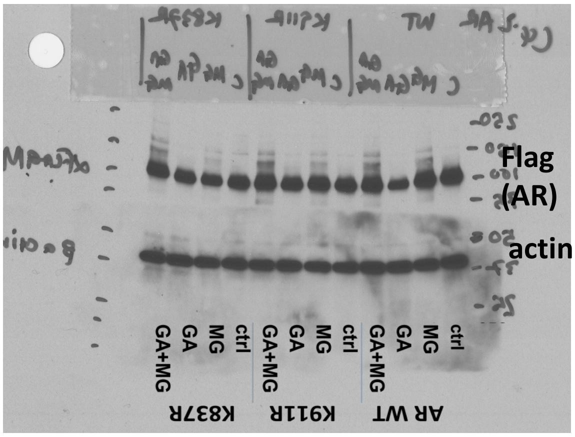

Supplementary Figure 10. Gels corresponding to Figure 4. Molecular weights were determined by overlaying autorads onto membranes with colored markers and marking band positions.

Figure 5c

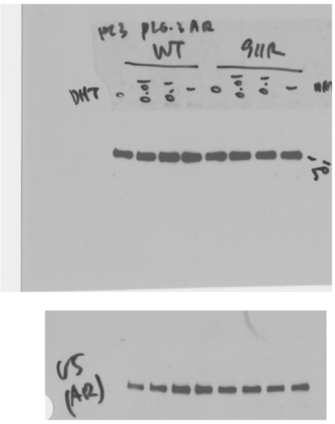

tubulin

V5
(AR)

Figure 5e

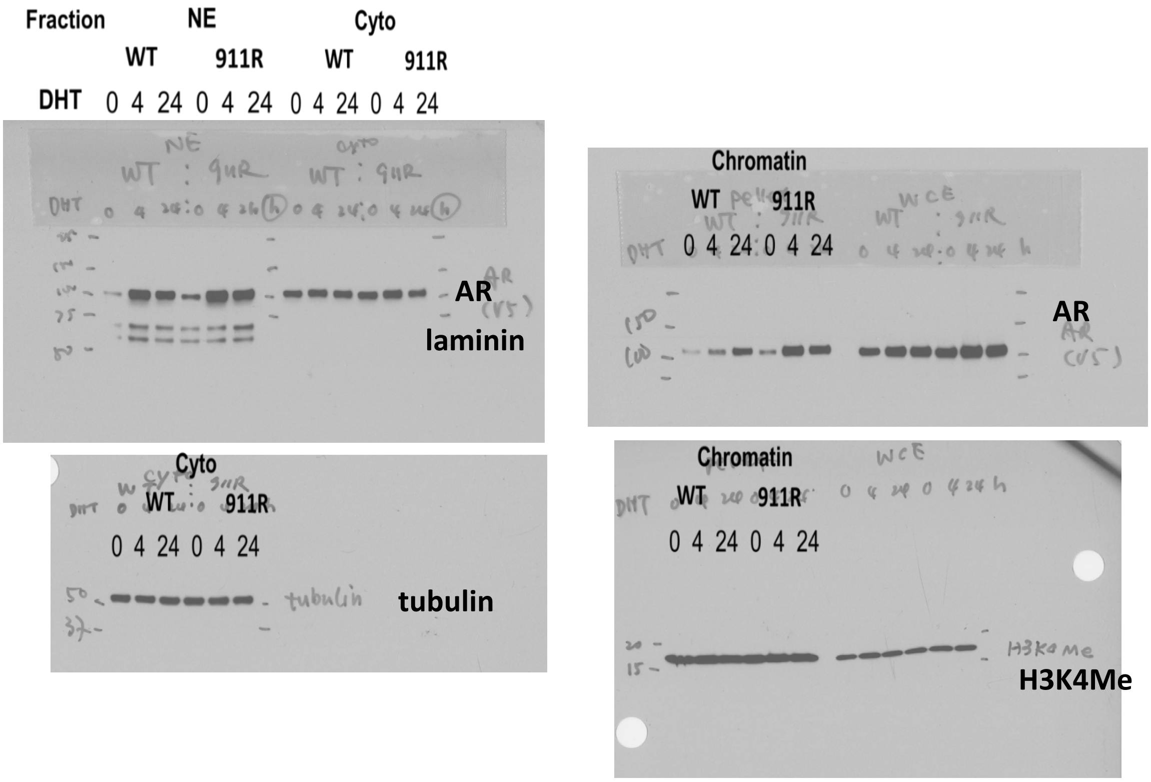

Supplementary Figure 11. Gels corresponding to Figure 5. Molecular weights were determined by overlaying autorads onto membranes with colored markers and marking band positions.

Figure 6b

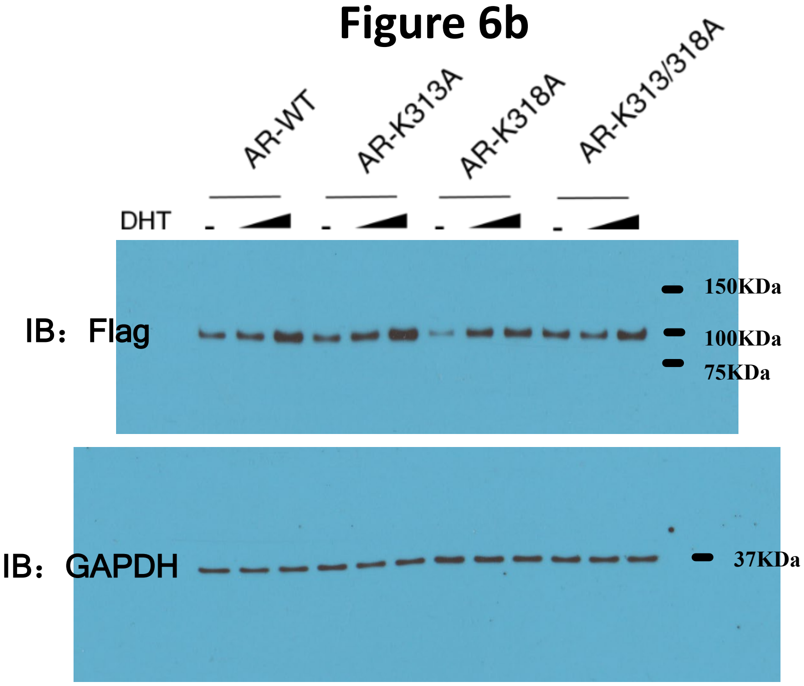

Figure 6d

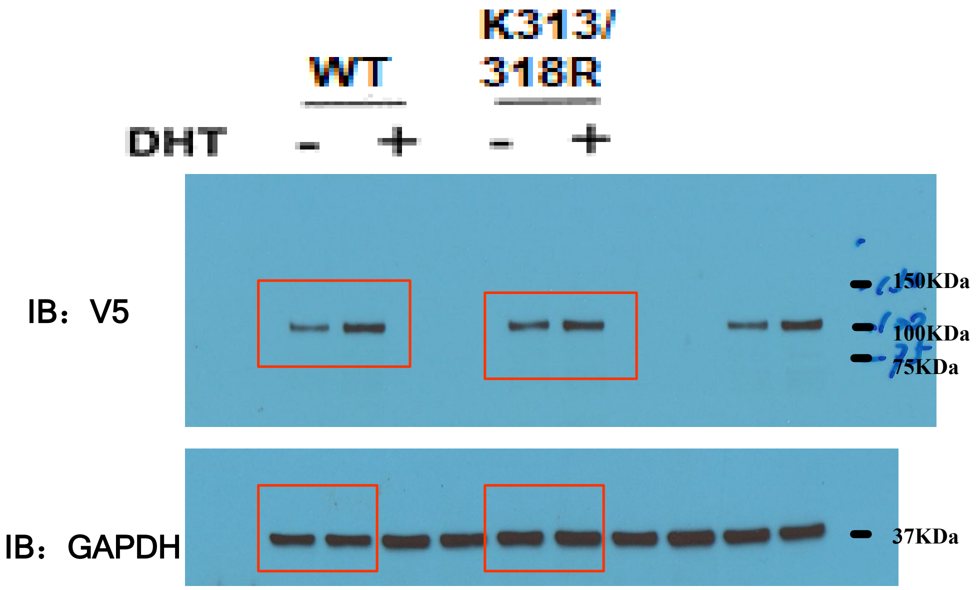

Supplementary Figure 12. Gels corresponding to Figure 6. Portions of the gel shown in the figure are highlighted. Molecular weights were determined by overlaying autorads onto membranes with colored markers and marking band positions.

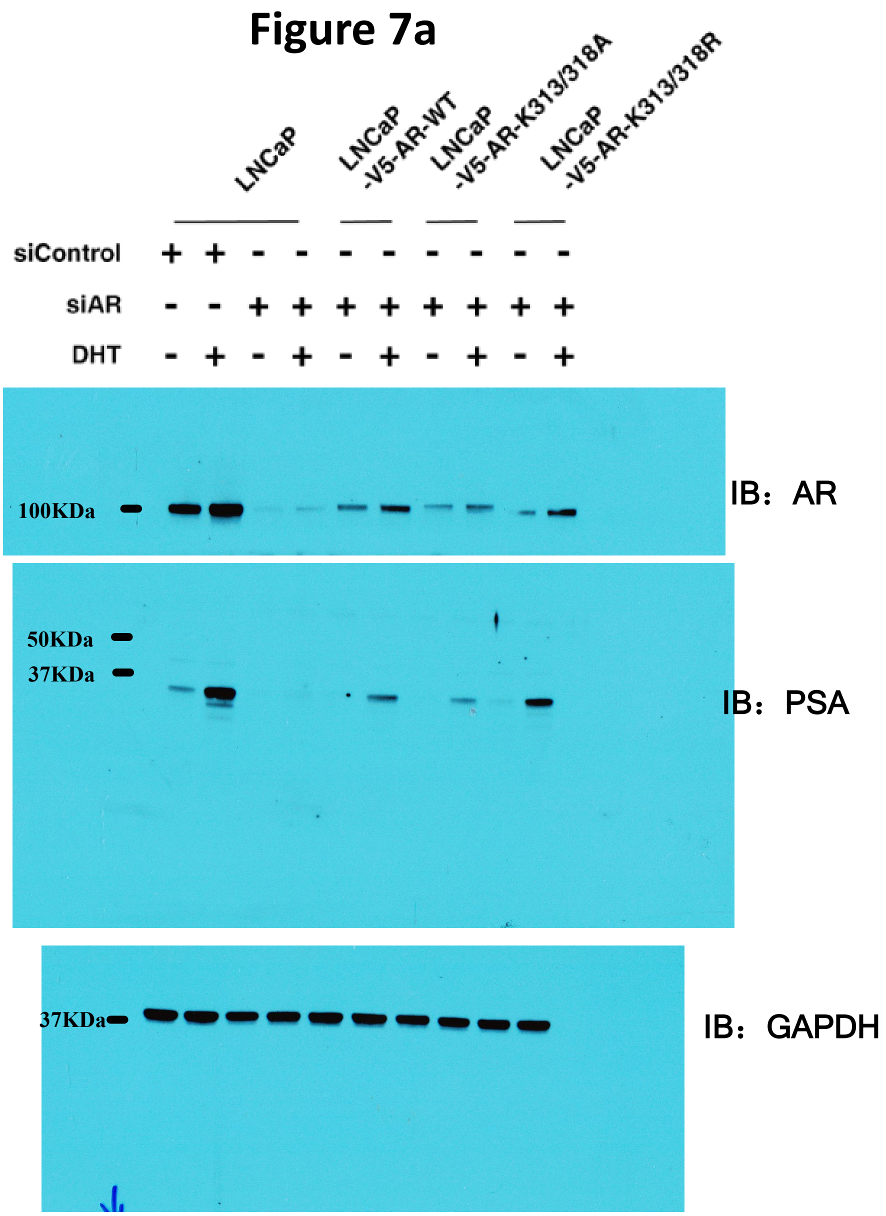

Supplementary Figure 13. Gel corresponding to Figure 7. Molecular weights were determined by overlaying autorads onto membranes with colored markers and marking band positions.

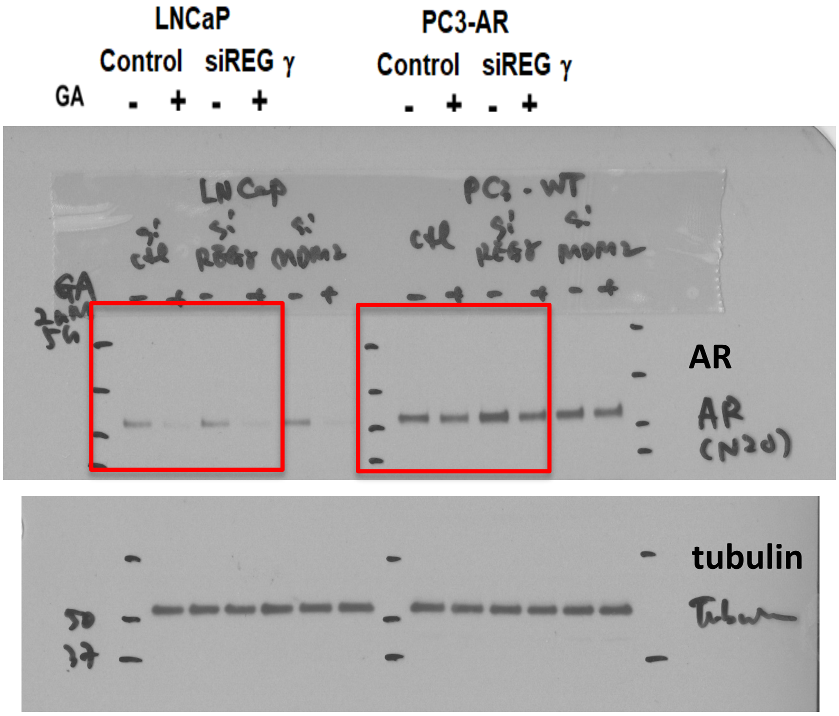

Supplementary Figure 14. Gel corresponding to Supplementary Figure 1. Portions of the gel shown in the figure are highlighted. Molecular weights were determined by overlaying autorads onto membranes with colored markers and marking band positions.

Supplementary Figure 2a

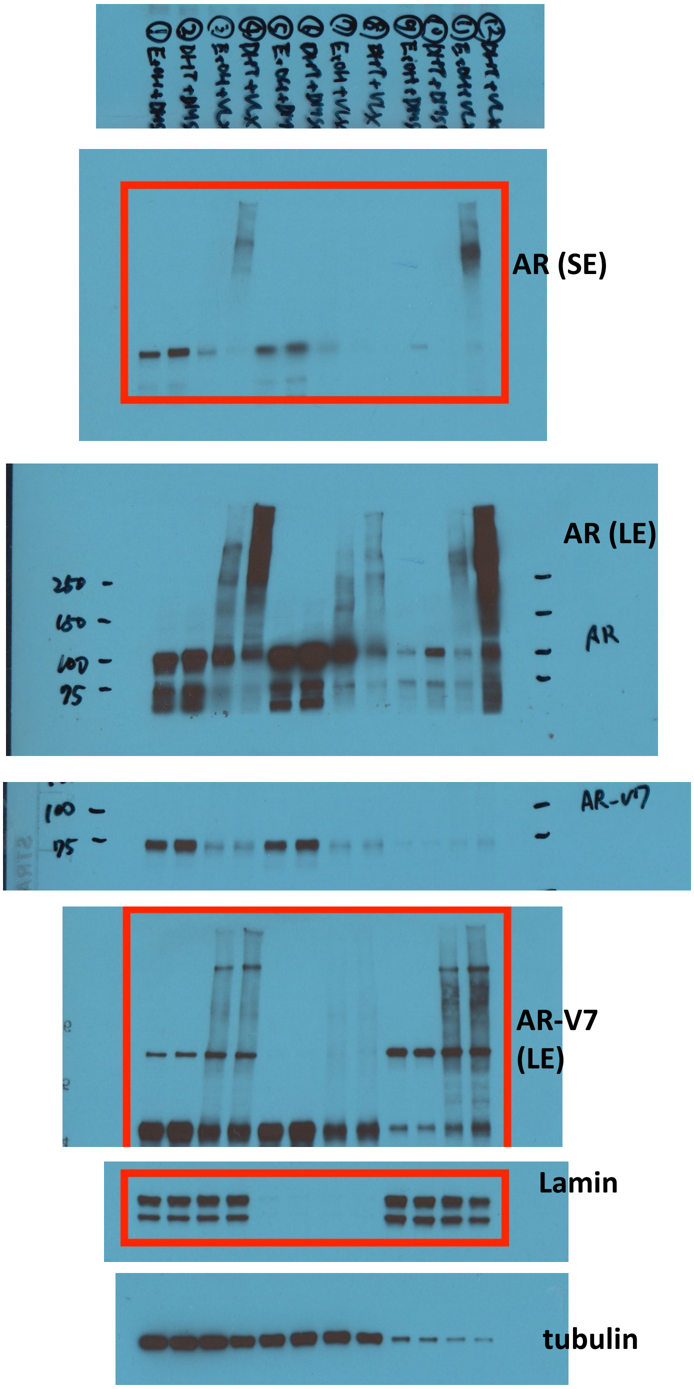

Supplementary Figure 2b

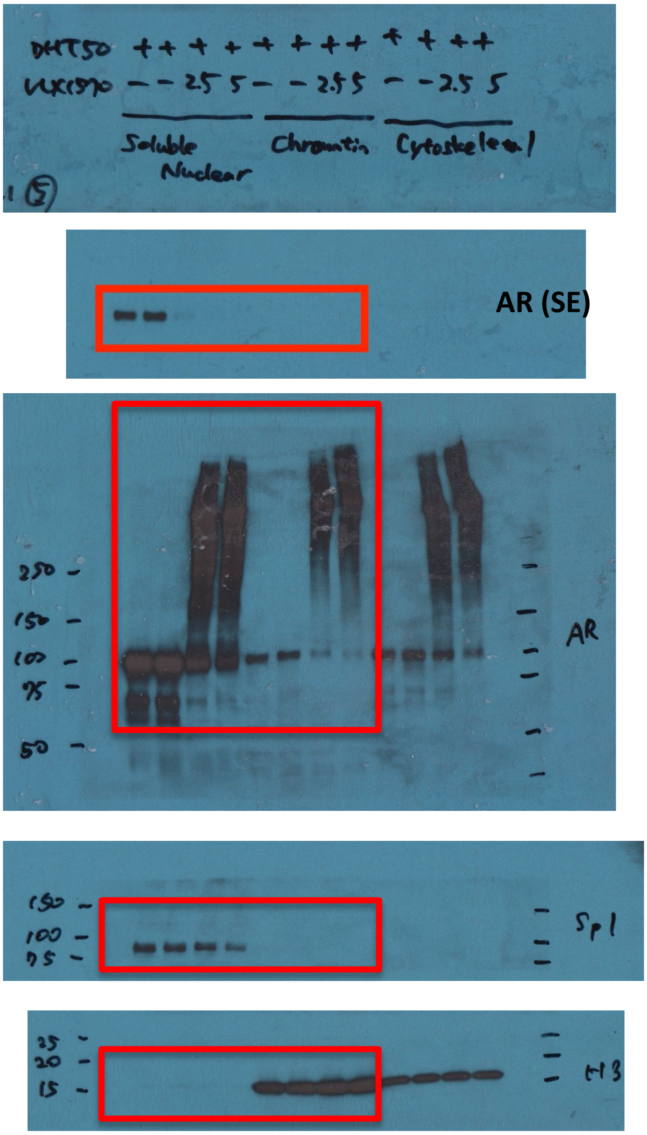

Supplementary Figure 15. Gels corresponding to Supplementary Figure 2. Portions of the gel shown in the figure are highlighted. Molecular weights were determined by overlaying autorads onto membranes with colored markers and marking band positions.

Supplementary Figure 3a

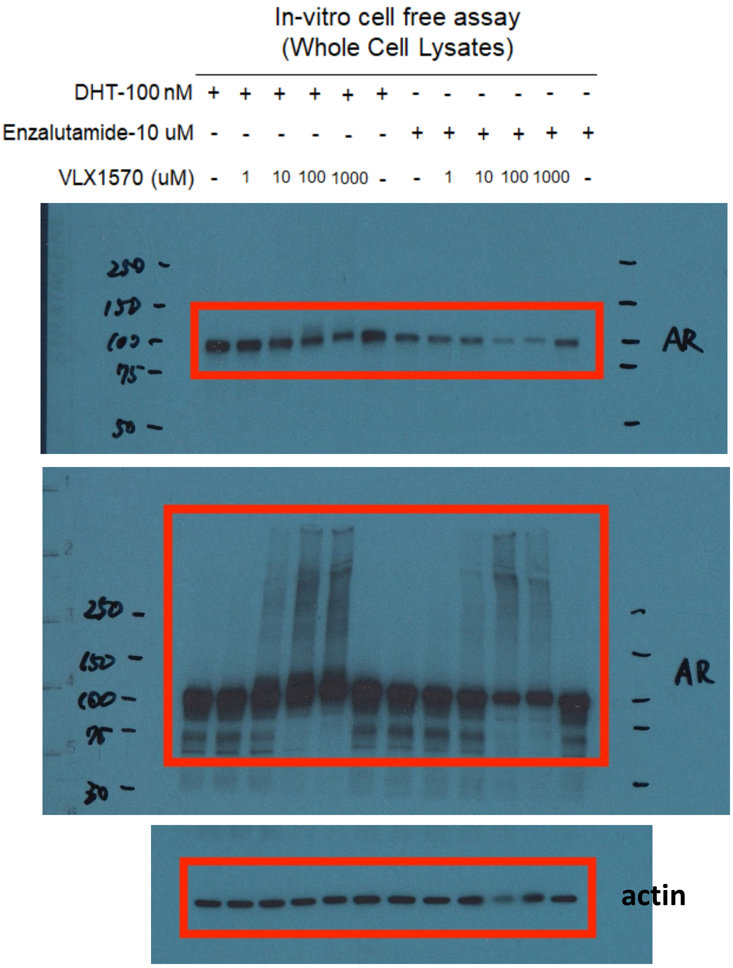

Supplementary Figure 3b

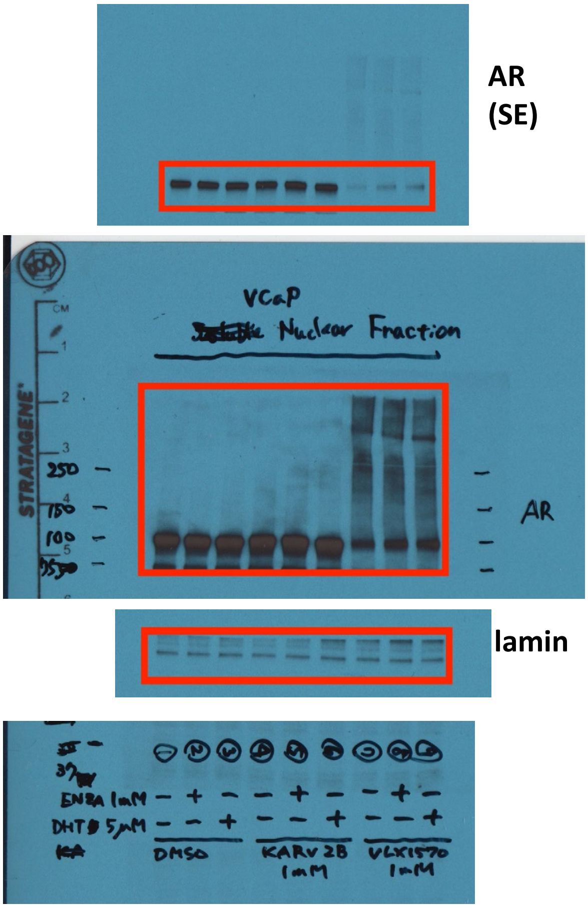

Supplementary Figure 16. Gels corresponding to Supplementary Figure 3. Portions of the gel shown in the figure are highlighted. Molecular weights were determined by overlaying autorads onto membranes with colored markers and marking band positions.

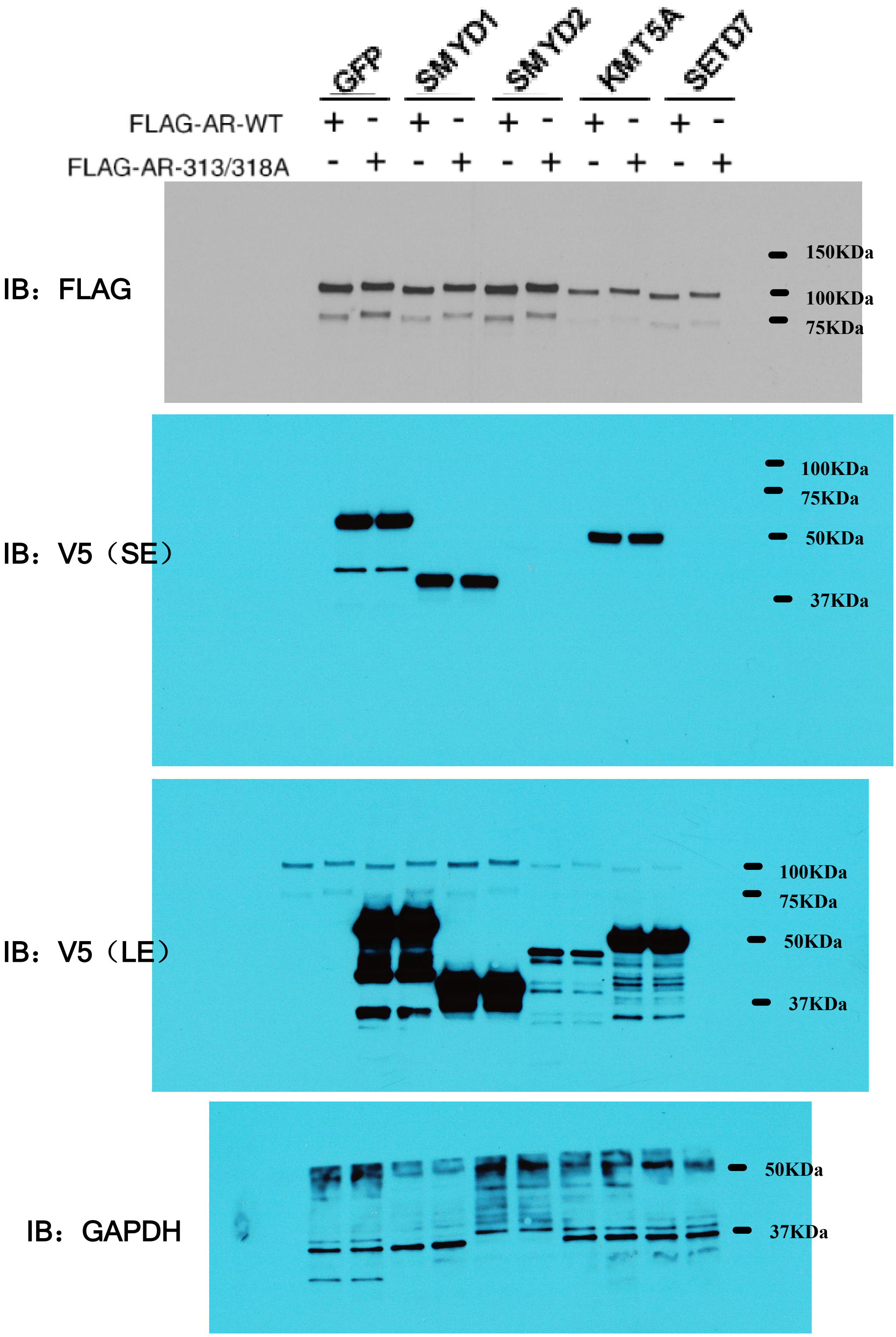

Supplementary Figure 17. Gel corresponding to Supplementary Figure 5b. Molecular weights were determined by overlaying autorads onto membranes with colored markers and marking band positions.

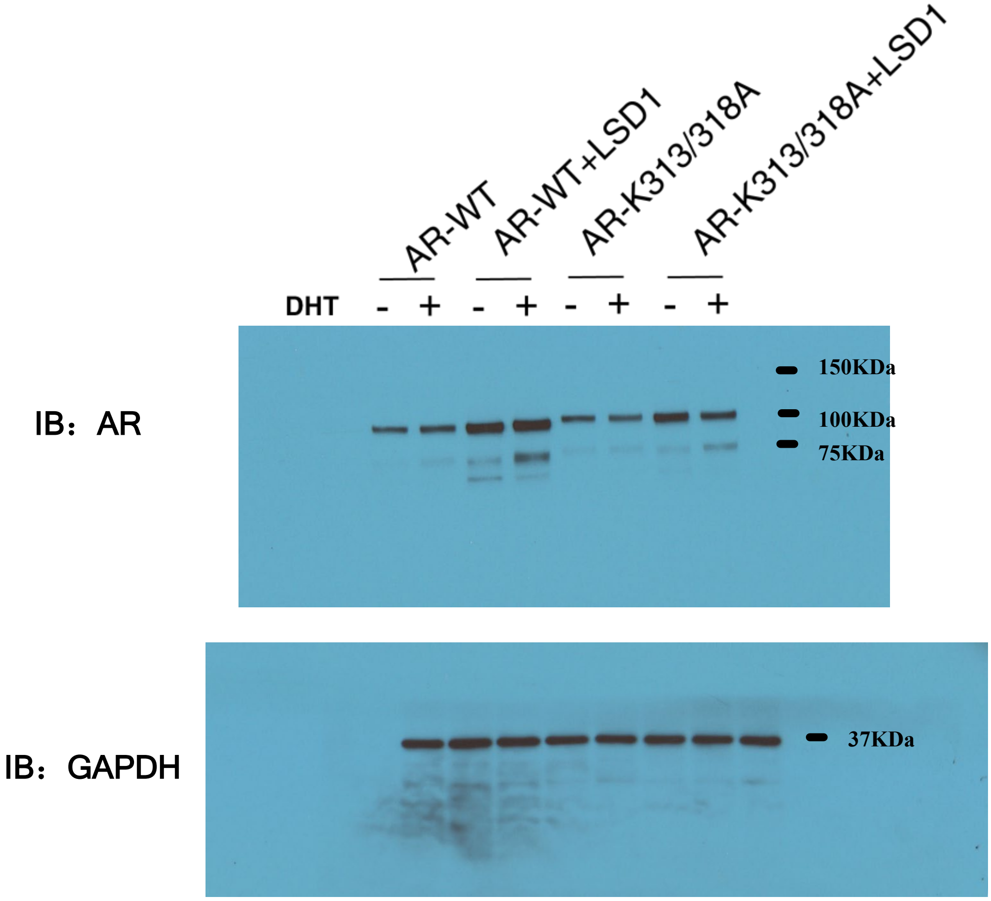

Supplementary Figure 18. Gel corresponding to Supplementary Figure 6b. Molecular weights were determined by overlaying autorads onto membranes with colored markers and marking band positions.
