# Supplementary material for: A carboxy-terminal ubiquitylation site regulates androgen receptor activity
Source: Commun Biol. 2024 Jan 5;7:25. doi: 10.1038/s42003-023-05709-x (PMC10770046; doi:10.1038/s42003-023-05709-x)
Supplement: Supplementary file 3 — Description of Additional Supplementary Files [file 42003_2023_5709_MOESM3_ESM.pdf]

Description of Additional Supplementary Files

File name: Supplementary Data 1
Description: Source data for Figures 5, 6, and 7, and Supplementary Figures 5 and 6.
